# *Chironomus ramosus* Larval Microbiome Composition Provides Evidence for the Presence of Detoxifying Enzymes

**DOI:** 10.3390/microorganisms9081571

**Published:** 2021-07-23

**Authors:** Rotem Sela, Sivan Laviad-Shitrit, Leena Thorat, Bimalendu B. Nath, Malka Halpern

**Affiliations:** 1 Department of Evolutionary and Environmental Biology, University of Haifa, Haifa 3498838, Israel; rotemselaf@gmail.com (R.S.); sivanlaviad@gmail.com (S.L.-S.); 2 Department of Zoology, Savitribai Phule Pune University, Pune 411007, India; leenathorat@gmail.com (L.T.); bbnath@gmail.com (B.B.N.); 3 Department of Biology, York University, Toronto, ON M3J 1P3, Canada; 4 Department of Biology and Environment, Faculty of Natural Sciences, University of Haifa, Oranim, Tivon 3600600, Israel

**Keywords:** chironomid, microbiome, host–bacteria interaction, toxicant, degradation resistance

## Abstract

Chironomids (*Diptera*; *Chironomidae*) are aquatic insects that are abundant in freshwater. We aimed to study the endogenous microbiota composition of *Chironomus ramosus* larvae that were sampled from the Mutha River and a laboratory culture in India. Furthermore, we performed a metagenomic analysis of the larval microbiome, sampled from the Mutha River. Significant differences were found between the bacterial community composition of *C. ramosus* larvae that were sampled from the Mutha River and the laboratory culture. A total of 54.7% of the amplicon sequence variants (ASVs) that were identified in the larvae from the Mutha River were unique, compared to only 12.9% of unique ASVs that were identified from the laboratory-reared larvae. The four most abundant phyla across all samples were: *Proteobacteria*, *Fusobacteria*, *Firmicutes*, and *Bacteroidetes*, while the nine most abundant genera were: *Aeromonas*, *Alkanindiges*, *Breznakia*, *Cetobacterium*, *Chryseobacterium*, *Desulfovibrio*, *Dysgonomonas*, *Thiothrix*, and *Vibrio*. Moreover, in the metagenomic analysis, we detected bacterial genes and bacterial pathways that demonstrated the ability to degrade different toxic compounds, detoxify metal, and confer resistance to antibiotics and UV radiation, amongst other functions. The results illuminate the fact that there are detoxifying enzymes in the *C. ramosus* larval microbiome that possibly play a role in protecting the insect in polluted environments.

## 1. Introduction

Members of the genus *Chironomus* (*Diptera*; *Chironomidae*) are aquatic insects. Their life cycle consists of complete metamorphosis. Three stages develop in the water, while the adults are aerial. *Chironomus* females lay egg masses inserted in a viscous substance at the water’s edge or on floating plants [1]. After hatching, the larva swims to the bottom of the water body to create a silken tube [1,2]. *Chironomus* larvae contain hemoglobin which enables them to survive at low oxygen concentrations [3,4,5]. The larvae exhibit tolerance to various toxic or stressful environmental conditions such as temperature, pH, salinity, dehydration, ultraviolet light (UV), toxic substances, and gamma radiation [1,6,7,8,9,10].

In a recent study, almost half of the microbial community that was identified in *C. transvaalensis* egg masses belonged to bacterial genera that might have the ability to protect the insect under stress conditions [11]. The first instar larva feeds on the egg mass remains which contain both nutrients and endogenous microorganisms [12]. It is hypothesized that the microbiota that inhabits the egg mass protects the insect along its metamorphosis [11]. Boush and Matsumura [13] were the first to provide evidence that insects’ microbiota can protect their host from pesticides. They showed that *Pseudomonas melophthora* that was isolated from the apple maggot (*Rhagoletis pomonella*) can degrade organophosphate pesticides and thus protect the insect [13]. Resistance of insects to pesticides has become a problem since insects’ symbiont communities evolve quickly to contain bacterial species that demonstrate the ability to detoxify pesticides [14].

A recent study of the *C. transvaalensis* microbiota composition of the four developmental life stages found that the microbiota composition changed throughout metamorphosis [15]. The researchers also showed that the microbiota composition was life stage-specific. Thus, this microbiota probably plays a role in supporting the insect’s development and survival [15]. Nevertheless, a microbiota core was always present in all life stages [15]. In an experimental setup, Senderovich and Halpern (2013) demonstrated that the endogenous microbiota of *C. transvaalensis* in the larval stage protects the larvae from toxic heavy metals such as hexavalent chromium and lead [6]. Halpern and Senderovich (2015) suggested that the diverse arsenal of bacteria that inhabit chironomids defend the insect under different stress conditions [1]. Recently, we showed that endogenous bacterial communities that inhabit *C. transvaalensis* support their host’s survival in a toxic environment containing copper and chromium [8].

Here, we hypothesize that the microbial community of *C. ramosus* larvae plays a role in protecting its host under toxic conditions, as demonstrated for *C. transvaalensis* [6,8]. In the current study, we explored, for the first time, the microbiota composition of *C. ramosus* larval samples from a river and a laboratory culture from India. In addition, using metagenomics, we found evidence of the presence of genes in the microbiome that encode resistance to toxic substances and polluted conditions.

## 2. Materials and Methods

**Chironomid sampling**. Chironomid larvae were sampled in November 2018 from (i) the Mutha River, Pune, India (18.2901° N, 73.4956° E), and (ii) a laboratory culture which was initially collected from the Mula River, Pune, India (18.5551° N, 73.8618° E), in April 2018, and maintained under laboratory conditions for eight months until it was sampled for our current study. The sampling procedure and the conditions for rearing the laboratory culture were previously described, in detail, by Laviad-Shitrit et al. [16]. In total, 31 larvae that were identified as *C. ramosus* were selected for further analyses in the current study (Supplementary Table S1).

To remove bacteria that were not tightly attached to the larval samples, immediately after sampling, each larva was washed and vortexed for one min in 1 mL sterile saline water (0.85% NaCl). The procedure of washing and vortexing was repeated five times. This technique was previously shown to reliably remove bacteria that do not belong to the insect’s endogenous bacterial microbiota [17]. Samples were kept in 2 mL of 95% ethanol at −20 °C.

**DNA extraction**. Each individual larva was homogenized separately in sterile saline water, and DNA was extracted using a DNA isolation kit (DNeasy Blood and Tissue, Qiagen, Germany), as described previously [18]. No DNA was detected in the NanoDrop One/OneC Spectrophotometer (Thermo Fisher Scientific, Waltham, MA, USA) when DNA extraction was performed using the DNA isolation kit on three blank samples that contained only sterile saline water but with no addition of a larva. The extracted DNA was stored at −20 °C.

**Chironomus species identification**. To identify chironomid species, we sequenced the cytochrome oxidase subunit I gene, in accordance with Folmer et al. [19]. The cytochrome oxidase subunit I gene sequences were assessed using the National Center for Biotechnology Information (NCBI) BLAST engine (https://www.ncbi.nlm.nih.gov, accessed on 1 July 2021) and demonstrated more than 99% sequence similarity to *C. ramosus*. Sequences were deposited in the NCBI GenBank database under the accession numbers MN934225-MN934227; MN934229-MN934232; MN93434-MN93436; MN93438-MN934239; MN934241; MN934243; MN934245-MN934249; MN934251-MN934254; MN934275; MN934285-MN934286; MN934291; MN934293; MN934300-MN9343001; MN934313.

**Analysis of the microbiota composition**. Genomic DNA was prepared for sequencing by employing a two-stage amplicon sequencing workflow, as described previously [15,20]. The 515F and 806R primers used targeted the V4 region of the 16S rRNA genes. The primers were synthesized with 5′ linker sequences compatible with Access Array primers for Illumina sequencers [21] (Sigma-Aldridge, Israel): CS1_515F: 5’-ACACTGACGACATGGTTCTACAGTGCCAGCMGCCGCGGTAA-3’; and CS2_806R: 5’-TACGGTAGCAGAGACTTGGTCTGGACTACHVGGGTWTCTAAT-3’. The PCR procedure was described, in detail, in Sela et al. [15]. Sterile DNA-free water was used as a negative control to verify the absence of contamination. No contamination was detected.

To incorporate Illumina sequencing adapters and a sample-specific barcode, a second PCR amplification was performed, as described in Sela et al. [15]. Each sample received a separate primer pair with a unique 10-base barcode, obtained from the Access Array Barcode Library for Illumina (Item# 100-4876, Fluidigm, South San Francisco, CA, USA). Pooled libraries, with a 20% phiX spike-in, were loaded onto an Illumina MiniSeq mid-output flow cell employing paired-end 2 × 150 base reads. The second-stage PCR amplification, pooling, and sequencing were conducted at the Genome Research Core (GRC) at the University of Illinois at Chicago (UIC).

**Sequence analysis**. One hundred and twenty-four fastq files were created, corresponding to 31 samples (four files for each sample), with two paired-end sequence sets each. Bioinformatic analysis was performed with the DADA2 pipeline (DADA2 v.1.14.0) [22]. First, raw sequences were quality filtered, trimmed (maxN = 0, maxEE = 2, trimLeft = 15bp, truncLen = 150), and merged (min overlap = 8bp). Next, both runs were merged by sample (resulting in 31 fastq format files) and checked for chimeras using the DADA2 pipeline, as described in Laviad-Shitrit et al. [8]. In total, for the 31 samples, 2,431,002 sequences (2423–177,570 per sample) were binned into 817 ASVs. To overcome the bias of an imbalanced sequencing depth, ASV relative abundances per sample were used for downstream analysis. The raw sequence data were deposited in the NCBI SRA repository (https://www.ncbi.nlm.nih.gov/sra (accessed on 1 July 2021)) under accession number PRJNA678128.

**Statistical analysis**. The mean relative abundances of the phyla and genera for each sampling site were calculated after combining ASVs based on taxonomic identification at each level. The most abundant phyla (over 4% of the reads) and genera (over 1% of the reads) were selected and presented in a column graph created by Microsoft Excel 2019. ASVs that were uniquely present in each of the places or that were shared between them were evaluated using the InteractiVenn online tool [23]. To study differences in the microbial community composition among *C. ramosus* populations between the two environments (Mutha River vs. laboratory), a non-metric multidimensional scaling analysis (NMDS), based on UniFrac (weighted) distance matrices, was performed using MicrobiomeAnalyst (http://www.microbiomeanalyst.ca (accessed on 1 July 2021)) [24]. The phylogenetic tree used in the Unifrac was constructed using Silva (www.arb-silva.de (accessed on 1 July 2021)) [25]. Analysis of similarities (ANOSIM) between the samples from the Mutha River and the laboratory culture was performed at three levels (ASVs; genera; phyla), using PRIMER v.7. [26]. Bacterial richness (Chao1) and diversity (Shannon) were calculated after the samples were rarefied for the low number of sequences (2423) using the MicrobiomeAnalyst tool. The richness and diversity parameters were compared by non-parametric tests (Kruskal–Wallis), followed by post hoc tests (Mann–Whitney) with Bonferroni correction for multiple comparisons (IBM SPSS v.25.0.0.1). Visualization was performed with Microsoft Excel 2019.

**Metagenomic analyses**. Three *C. ramosus* larval samples that were sampled from the Mutha River were chosen for metagenomic analyses. For DNA metagenomic sequencing, we used 100 ng genomic DNA. Sample preparation and sequencing procedures were described in Laviad-Shitrit et al. [8]. Library preparation, quality control sequencing, and Nova-Seq 6000 sequencing were performed at the DNA Services Laboratory at the University of Illinois at Urbana-Champaign. Metagenomic sequences were deposited in the NCBI Sequence Read Archive (SRA) (https://www.ncbi.nlm.nih.gov/Traces/study/?acc=PRJNA604900 (accessed on 1 July 2021)) as BioProject PRJNA604900.

**Taxonomic profiling**. Raw reads were mapped to the NCBI nucleotide database using the Centrifuge engine [27]. Taxonomic annotations for every read were obtained using the least common ancestor algorithm. Then, raw counts were standardized to percentages for relative abundance. The metagenomic results included both bacterial and eukaryote sequences. The sequence analysis that was performed in the current research focused only on the sequences that were classified as belonging to the domain Bacteria.

**Functional profiling**. Raw reads were mapped to the Swissprot protein database using DIAMOND [28,29]. Gene ortholog annotations were assigned using the consensus of aligned references and then summarized across all reads to create counts per ortholog for each sample. Higher-level summaries of orthologous functions were created using KEGG BRITE hierarchical annotations [30]. Raw counts were standardized to percentages of relative abundance.

## 3. Results

**Chironomid species identification**. Chironomid larvae were sampled from the Mutha River and a laboratory culture in Pune, India. The larval species was identified by sequencing the cytochrome oxidase subunit I gene. BLAST analysis of the sequences revealed that 23 larvae that were sampled from the Mutha River and 8 larval samples from the laboratory culture belonged to the *C. ramosus* species (with more than 99% sequence similarity).

**Figure 1:**
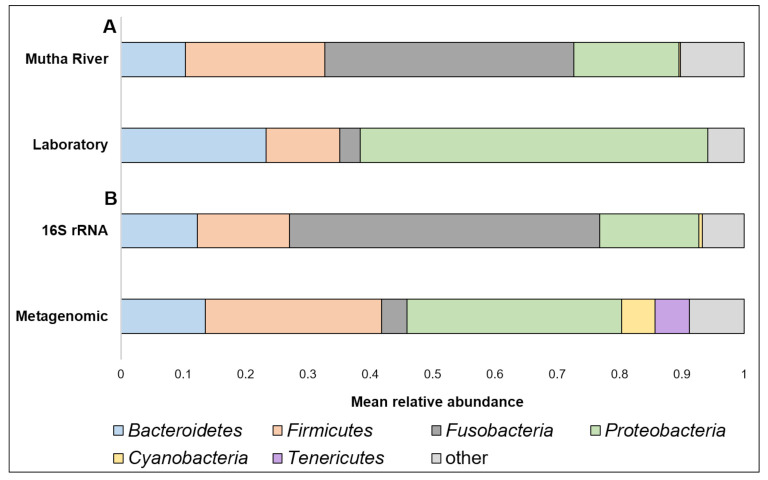
(**A**) A comparison between the mean relative abundance (>4% of the reads) of *C. ramosus* larval microbiota composition at the phylum level between larvae that were sampled from the Mutha River and the laboratory culture. (**B**) A comparison between the mean relative abundance (>4% of the reads) of *C. ramosus* larval microbiota composition at the phylum level between the three larval samples from the Mutha River that were analyzed using both 16S rRNA gene sequencing and metagenomic analysis.

**Taxonomy composition**. A detailed list of the ASVs’ identities in the different larval samples is presented in Supplementary Table S2. Overall, 22 phyla were detected. The four most abundant (>4% of the reads) across all samples were *Proteobacteria*, *Fusobacteria*, *Firmicutes*, and *Bacteroidetes* (Figure 1A). When the taxonomy of the three larvae sampled from the Mutha River was analyzed using metagenomics and compared to the 16S rRNA gene sequencing data, we detected two more phyla in high abundance: *Cyanobacteria* (5.3%) and *Tenericutes* (5.5%) (Figure 1B).

**Figure 2:**
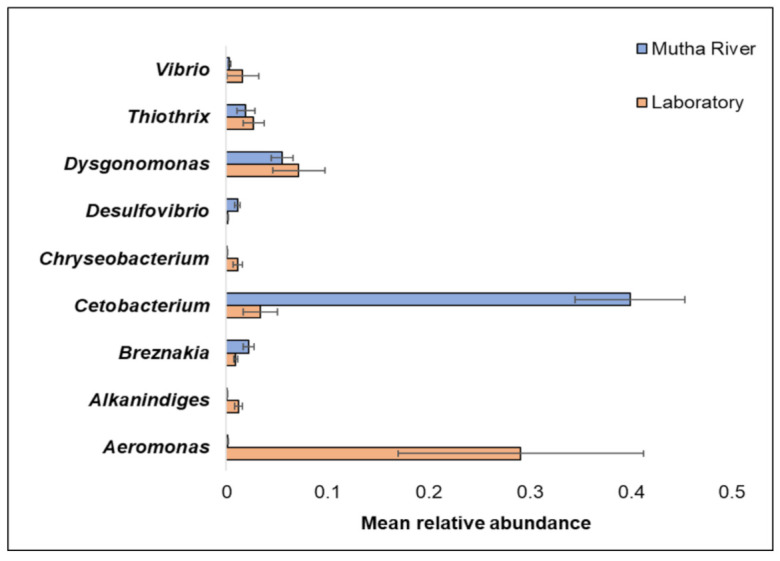
The mean relative abundance of nine prominent genera that were identified in the larval microbiota composition that were sampled from the two studied environments (Mutha River vs. laboratory). Bars represent mean ± standard error.

Overall, 200 genera were detected (Supplementary Table S2). The nine most abundant (>1% of the reads) across all samples in the 16S rRNA gene sequencing analysis were *Aeromonas*, *Alkanindiges*, *Breznakia*, *Cetobacterium*, *Chryseobacterium*, *Desulfovibrio*, *Dysgonomonas*, *Thiothrix*, and *Vibrio* (Figure 2). When the most abundant genera were extracted from the metagenomic data, 14 genera were detected (Supplementary Figure S1). The most abundant genera in the metagenomic analysis (>2% of the reads) were: *Mycoplasma*; *Fusobacterium*; *Pseudomonas*; *Campylobacter*; *Bacillus*; and *Clostridium* (Supplementary Figure S1). No significant differences were found between the *C. ramosus* larval bacterial community at the phyla and genera levels in the 16S rRNA data analysis, between the two habitats (laboratory vs. Mutha River) (ANOSIM phyla: R = −0.048, *p* = 0.63; ANOSIM genera: R = 0.19, *p* = 0.053).

**Figure 3:**
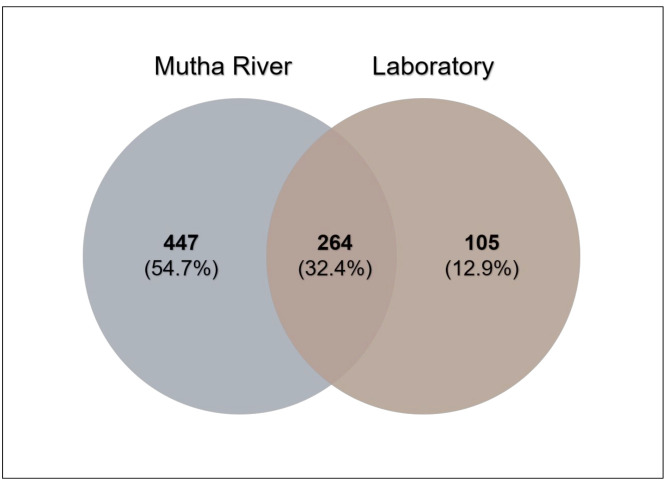
Venn diagram showing the overlap of *C. ramosus* larval bacterial ASVs across the two environments. The Venn diagram shows the percentage of the ASVs that are unique to each environment (Mutha River: 54.7%, laboratory: 12.9%), and the percentage of the ASVs that are shared (32.4%).

**Venn diagram** analysis (Figure 3) demonstrated the unique and shared ASVs between the two sampled habitats. More unique ASVs were found in the Mutha River larvae (54.7%) compared to the laboratory larval samples (12.9%). Interestingly, although larval samples from the laboratory were maintained for more than eight months in different conditions, about one-third (32.4%) of the ASVs were shared between the river and the laboratory samples.

**Figure 4:**
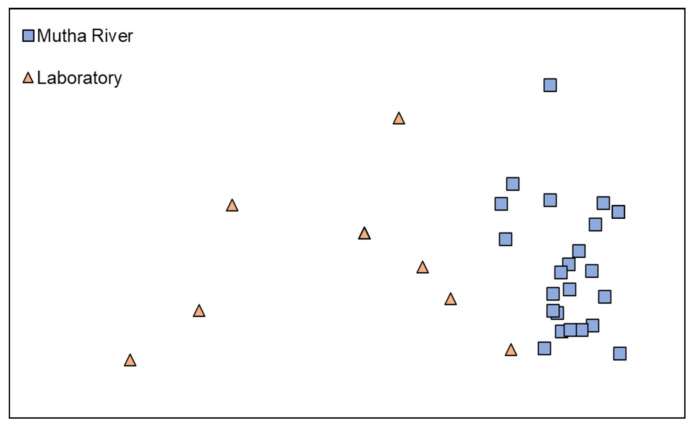
Non-metric multidimensional scaling (NMDS) analysis, representing the bacterial composition of *C. ramosus* larvae in the two sampling environments: Mutha River vs. laboratory. Stress = 0.09 (weighted UniFrac), *n* = 31 samples. Significant differences were found in the larval microbiota composition between the larvae that were sampled from the two sampling points (ANOSIM: R = 0.55, *p* < 0.001).

**Figure 5:**
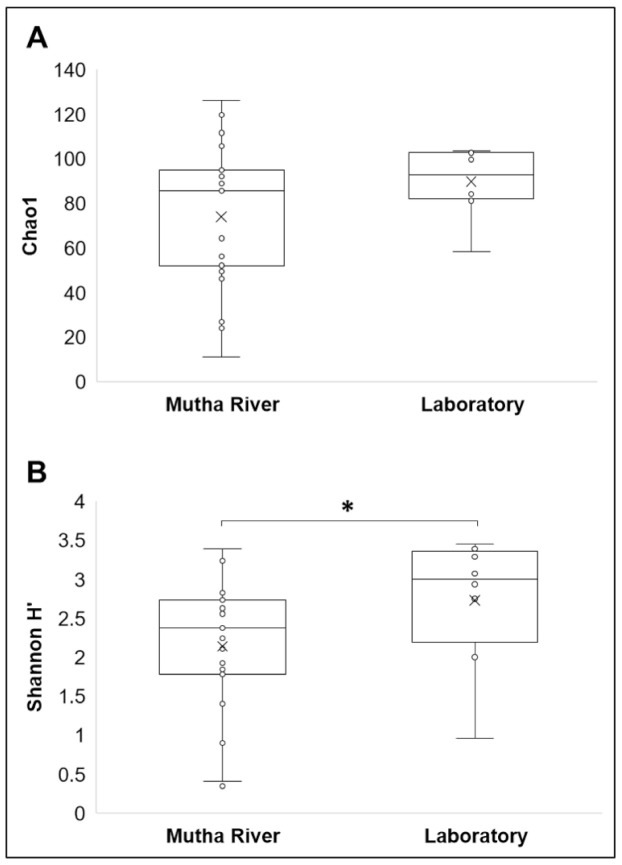
Alpha diversity of *C. ramosus* larval microbiota. The two estimators: (**A**) Chao1; (**B**) Shannon H’), were calculated after rarefying at the ASV level. No significant differences were found in the microbial richness (Chao1) between environmental and laboratory larval samples (Kruskal–Wallis: X1 = 1.486, *p* = 0.223), while the microbial diversity (Shannon H’) was significantly different between the microbiota from the two environments (Kruskal–Wallis: X1 = 4.696, *p* = 0.03). * Significant difference.

**Microbiota composition**. The NMDS plot (weighted UniFrac distance matrix, stress = 0.09) showed that the microbiota composition of *C. ramosus* larvae that were sampled from two different habitats (laboratory vs. Mutha River) clustered separately (Figure 4). Significant differences were found between *C. ramosus* larval bacterial community compositions that were sampled in the Mutha River and the laboratory culture (ANOSIM: R = 0.55, *p* < 0.001). No significant differences were found between the microbial richness (Chao1) of the larval microbiota that were sampled from the laboratory and the river (Kruskal–Wallis: X1 = 1.486, *p* = 0.223), while the bacterial diversity (Shannon H’) was significantly different (Kruskal–Wallis: X1 = 4.696, *p* = 0.03), with a higher index for the laboratory samples (Figure 5). The indices calculated for the microbiota composition of the river larval samples were more scattered compared to the laboratory larval samples (Figure 5).

**Metagenomic analysis**. To study the genes that are encoded by the endogenous bacteria that inhabit *C. ramosus* larvae, and, in particular, to find indications for the presence of bacterial genes that may play a role in assisting the larvae to survive in toxic and polluted environments, we performed a metagenomic analysis on three *C. ramosus* larval samples from a natural environment, the Mutha River.

**Figure 6:**
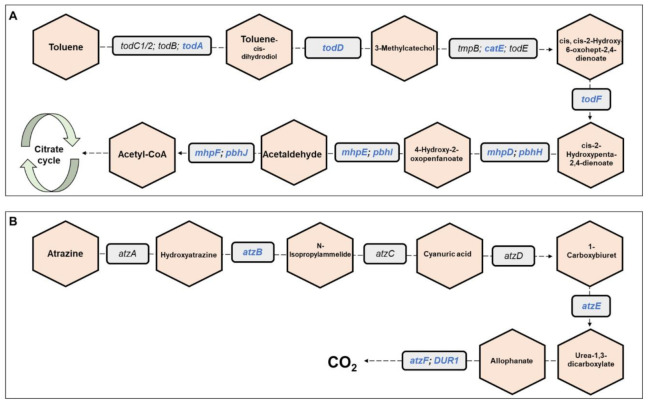
Toluene and atrazine degradation pathways. The figure presents a detailed example of: (**A**) the toluene degradation pathway, and (**B**) the atrazine degradation pathway (see also Table 1), which were found in the metagenomic data analysis of the three *C. ramosus* larvae. Genes belonging to these pathways and that were found in our metagenomic data analysis are highlighted in blue. Molecules are presented in hexagons; genes are presented in squares.

**Metagenomics pathways**. Overall, 445 bacterial functional pathways were detected. Evidence for biosynthesis of antibiotic compounds was found in 1.44% of the pathways. *Vibrio cholerae*, which causes cholera disease, was detected in 0.169% of the pathways (ko05110). Evidence for detoxifying pathways was also detected, and several are presented in Table 1. Examples of toluene and atrazine degradation pathways are presented, in detail, in Figure 6. In the toluene pathway, degraded toluene enters the citrate cycle (Figure 6A).

**Metagenomics functions**. Overall, 11,959 bacterial functional genes were detected. We found 49 bacterial genes that were correlated with metal detoxification. Moreover, we found genes associated with resistance to UV radiation, stress regulators, and degradation of different toxic compounds. Evidence for the presence of functions related to resistance to different antibiotics, toxic metals, including arsenical, copper, and zinc resistance (*arsH*, *copB*, *zraP*), and UV radiation is presented in Table 2.

## 4. Discussion

We studied the microbiota composition of *C. ramosus* larvae that were sampled from two different habitats, a river and a laboratory culture, using 16S rRNA gene sequencing and metagenomic analysis. In the metagenomics analysis, we identified a list of pathways that demonstrated the detoxifying abilities of the bacterial communities that inhabit the larvae. These demonstrate that the *C. ramosus* larval microbiome may enable the survival of chironomids in polluted environments.

*Proteobacteria* was the most abundant phylum in the current study. *Proteobacteria* is well documented in chironomid species [1,8,11,15] and is known as a highly abundant phylum in insects [31]. *Tenericutes*, which was found to be a dominant phylum in the metagenomic analysis, was never described in chironomids before. A study of the microbial community of mayflies (aquatic insects; *A. rusticus*, *Cinygmula*, and *Epeors* sp.,) found that *Tenericutes* was one of the five most prevalent phyla [32].

Chironomids were found to be natural reservoirs of *V. cholerae* and *Aeromonas* sp. [1]. Recently, *Vibrio* and *Aeromonas* were suggested to serve as *C. transvaalensis* symbionts [15]. Moreover, toxigenic *V. choerae* O1 and O139 were previously detected in *C. ramosus* larvae [16]. In the current study, we confirmed these findings by identifying *Vibrio* and *Aeromonas* in larvae that were sampled both from the laboratory culture and the Mutha River, using both 16S rRNA gene sequencing and metagenomics methods.

The relative abundance of the genus *Cetobacterium* was very high in the *C. ramosus* larval microbiota sampled from the Mutha River (39.9%) compared to the larval microbiota that were sampled from the laboratory culture (3.3%). This suggests that *Cetobacterium* may play a role in the survival of the insect in its natural environment. *Cetobacterium* was also identified in different freshwater fish species [33]. Many freshwater fish species feed on chironomid larvae, potentially explaining the origin of *Cetobacterium* in the fish gut; such a pattern was previously described for *V. cholerae* [34]. *Cetobacterium* was found to promote the synthesis of vitamin B12 [35], an essential molecule for the insect. The contribution of *Cetobacterium* to fish and insect larva health has yet to be explored.

The Venn diagram of shared ASVs between the larvae that were sampled from the different environments illustrates that although significant differences were found between the microbiota compositions of the larvae that were sampled from the different environments, 32.4% of the ASVs were still shared between the larval microbiota from the river and the laboratory. The bacterial diversity (Shannon H’) was significantly different between the larval microbiota that were sampled from the laboratory and the river, while no significant differences were found between the microbial richness. Inexplicably, the richness and the diversity were higher for the laboratory larval samples. It is possible that under optimal conditions, there are more options for a wider and diverse range of species richness. An arsenal of bacterial species inhabits chironomids [1]. Perhaps, in specific environmental conditions, different microorganism species can protect the host. Therefore, the most adaptive ones will relatively proliferate under different conditions, while others will be relatively less abundant. The change in the prevalence of a specific species in the microbiome could be a rapid response to an environmental change [1]. For example, the microbiota composition of *C. transvaalensis* larvae exposed to toxic copper or hexavalent chromium for six days changed significantly compared to the untreated control larvae [8]. Our results show an increase in bacterial diversity when larvae were transferred from the environment to laboratory conditions, demonstrating that without selective pressure from extreme environmental conditions, the entire bacterial arsenal in the larvae can proliferate.

In the current study, we were able to identify detoxifying pathways stemming from the *C. ramosus* larval microbiome. Chironomids can tolerate a broad scale of stress situations, and it was suggested that their microbiota play a role in protecting them under these conditions [1,6,8]. Until now, this hypothesis remained unsubstantiated using molecular tools, and here, for the first time, we show that there are genes in the insects’ microbiota that protect them from various threats. In the metagenomic data, we were able to detect genes related to stress tolerance. An example of one of these genes is *arsH*, which encodes an arsenical resistance protein ArsH. This gene was identified in several bacterial species such as *Pseudomonas* sp. [36]. Indeed, *Pseudomonas* was identified in our data [1,8]. Moreover, *Pseudomonas* was also found to degrade pyrethrin and was identified among the gut microbiota of the oriental cockroach, *Blatta orientalis* [37].

One of the pathways that was detected in the larval microbiome is the toluene degradation pathway. Toluene is a monoaromatic, toxic compound. It is widely used as a chemical substance in various industrial processes; therefore, toluene is classified as an environmental contaminant [38]. Toluene can cause damage to the central nervous system [39]. The *Bacillus* and *Pseudomonas* species can degrade toluene and use it as a sole carbon source [40]. These bacterial genera were detected in high abundances in the metagenomic analysis.

Another pathway that was detected in the larval microbiome is atrazine degradation. Atrazine is used in agriculture for controlling the growth of grasses and braid-leaved weeds [41]. It is the most widely used herbicide in the world [42] and thus present in different water bodies around the world [41]. High concentrations of atrazine can be found in river estuary sediments [41], which chironomids harbor. The presence of the atrazine degradation pathway in the *C. ramosus* microbiome may illuminate the fact that atrazine is not toxic to midges [43]. A variety of Gram-negative and Gram-positive bacterial species were reported to degrade atrazine, including *Pseudomonas* [44], *Rhodococcus* [45], *Acinetobacter* [46], and *Arthrobacter* [47]. Indeed, the presence of these bacterial species in the metagenomic data was confirmed. The prevalence of *Acinetobacter* and *Pseudomonas* was relatively high. The dioxin degradation pathway was also observed in the *C. ramosus* larval microbiome, which indicates that *C. ramosus* is resistant to this toxic molecule. A study of *C. riparius* as ecotoxicity indicators of dibenzo-p-dioxins and dibenzofurans showed that there were no significant differences between larvae that were exposed to dioxin and untreated control larvae [48].

## 5. Conclusions

There are significant differences between the microbiota composition of *C. ramosus* larvae that were sampled from a river and a laboratory culture. As far as we know, this is the first study of the *C. ramosus* larval microbiota and microbiome composition. The results provide evidence for the presence of detoxifying enzymes and pathways in the larval microbiome of *C. ramosus*. The bacterial community composition likely plays a role in promoting the insect’s survival. Indeed, we detected pathways and functions in the *C. ramosus* microbiome that degrade, tolerate, and resist toxic compounds. We conclude that the chironomids’ endogenous microbiota enables the insect to live and survive under extreme environmental conditions. However, more molecular studies are required to better understand the mechanisms that enable these host–microbiota interactions.

## Figures and Tables

**Table 1:** The presence of detoxifying pathways that were identified in the metagenomic analysis of the *C. ramosus* larval microbiome.

Category	Id (Detoxifying Pathways)
ko00633	Nitrotoluene degradation
ko00623	Toluene degradation
ko00622	Xylene degradation
ko00642	Ethylbenzene degradation
ko00362	Benzoate degradation
ko00627	Aminobenzoate degradation
ko00364	Fluorobenzoate degradation
ko00945	Stilbenoid, diarylheptanoid, and gingerol biosynthesis
ko00626	Naphthalene degradation
ko00627	Aminobenzoate degradation
ko00364	Fluorobenzoate degradation
ko00625	Chloroalkane and chloroalkene degradation
ko00361	Chlorocyclohexane and chlorobenzene degradation
ko00642	Ethylbenzene degradation
ko00643	Styrene degradation
ko00791	Atrazine degradation
ko00930	Caprolactam degradation
ko00363	Bisphenol degradation
ko00621	Dioxin degradation
ko00624	Polycyclic aromatic hydrocarbon degradation
ko00365	Furfural degradation
ko00984	Steroid degradation
ko00980	Metabolism of xenobiotics by cytochrome P450
ko00982	Drug metabolism—cytochrome P450
ko00983	Drug metabolism—other enzymes

**Table 2:** The presence of bacterial functions that are related to resistance to antibiotics, toxic metals, and UV radiation, identified in the metagenomic analysis of the *C. ramosus* larval microbiome.

Category	Gene	Function (Resistance)
K07665	*cusR*, *copR*, *silR*	two-component system, OmpR family, copper resistance phosphate regulon response regulator CusR
K03327	TC.MATE, SLC47A, *norM*, *mdtK*, *dinF*	multidrug resistance protein, MATE family
K03297	*emrE*, *qac*, *mmr*, *smr*	small multidrug resistance pump
K08163	*mdtL*	MFS transporter, DHA1 family, multidrug resistance protein
K18924	*ykkC*	paired small multidrug resistance pump
K11741	*sugE*	quaternary ammonium compound resistance protein SugE
K03712	*marR*	MarR family transcriptional regulator, multiple antibiotic resistance protein MarR
K11811	*arsH*	arsenical resistance protein ArsH
K07245	*pcoD*	copper resistance protein D
K07156	*copC*, *pcoC*	copper resistance protein C
K21740	*rclB*	reactive chlorine resistance protein B
K02547	*mecR1*	methicillin resistance protein
K21252	*fosX*	fosfomycin resistance protein FosX
K07233	*pcoB*, *copB*	copper resistance protein B
K02617	*paaY*	phenylacetic acid degradation protein
K22011	*cfbA*	sirohydrochlorin cobalto/nickelchelatase [EC:4.99.1.3 4.99.1.11]
K07803	*zraP*	zinc resistance-associated protein
K15726	*czcA*	cobalt-zinc-cadmium resistance protein CzcA
K16264	*czcD*, *zitB*	cobalt-zinc-cadmium efflux system protein
K21903	*cadC*, *smtB*	ArsR family transcriptional regulator, lead/cadmium/zinc/bismuth-responsive transcriptional repressor
K00520	*merA*	mercuric reductase [EC:1.16.1.1]
K21903	*cadC*, *smtB*	ArsR family transcriptional regulator, lead/cadmium/zinc/bismuth-responsive transcriptional repressor

## Data Availability

The Illumina 16S rRNA raw sequence data and the metagenomic sequences were deposited in the NCBI SRA repository (https://www.ncbi.nlm.nih.gov/sra (accessed on 1 July 2021)) under the accession numbers PRJNA678128 and PRJNA604900, respectively. The cytochrome oxidase subunit I gene sequences that were used for the taxonomic identification of chironomid species were deposited in the NCBI GenBank database under the accession numbers: MN934225-MN934227; MN934229-MN934232; MN93434-MN93436; MN93438-MN934239; MN934241; MN934243; MN934245-MN934249; MN934251-MN934254; MN934275; MN934285-MN934286; MN934291; MN934293; MN934300-MN9343001; MN934313. Additional data are available on request from the corresponding author of this study.

## Supplementary Materials

The following are available online at https://www.mdpi.com/article/10.3390/microorganisms9081571/s1, Supplementary Table S1. A list of *C. ramosus* samples that were analyzed in the current study. The list of all ASVs is presented in Supplementary Table S2; Supplementary Table S2. ASV taxonomic classification and relative abundance for each sampled larva. Larvae were sampled from a laboratory culture and from the Mutha River. This table is presented in an accompanying Excel file; Supplementary Figure S1. The most abundant genera (over 1% of the reads, except for *Vibrio* and *Aeromonas*) across all samples. Results are from the metagenomic data of the three larval samples from the Mutha River.
