# Supplementary material for: Chironomus ramosus Larval Microbiome Composition Provides Evidence for the Presence of Detoxifying Enzymes
Source: Microorganisms. 2021 Jul 23;9(8):1571. doi: 10.3390/microorganisms9081571 (PMC8398091; doi:10.3390/microorganisms9081571)
Supplement: Supplementary file 1 [file microorganisms-09-01571-s001.zip › Supplementary Material 20.07.21 RS_MH.pdf]

Supplementary Material

Chironomus ramosus larval microbiome composition provides evidence for the presence of detoxifying enzymes

Rotem Sela[1], Sivan Laviad-Shitrit[1] Leena Thorat [2,3], Bimalendu B. Nath[2] and Malka Halpern[1, 4]

[1]*Department of Evolutionary and Environmental Biology, University of Haifa, Haifa, Israel*

[2]*Department of Zoology, Savitribai Phule Pune University, Pune, India*

[3]*Department of Biology, York University, Toronto, Canada*

[4]*Department of Biology and Environment, University of Haifa, Oranim, Tivon, Israel*

Supplementary Table S1. A list of *C. ramosus* samples that were analyzed in the current study. The list of all observed ASVs is presented in Supplementary Table S2.

Laboratory	Mutha River	
LBBLC	LBP2A	
LBBLN	LBP2B	
LBBLO	LBP2C	
LCBLE	LBP2F	
LCBLH	LBP2H	
LDBLB	LBP2I	
LDBLC	LCP2A	
LDPLO	LCP2C	
	LCP2E	
	LCP2G	
	LCP2J	
	LCP2K	
	LCP2M	
	LCP2O	
	LDP2A	
	LDP2B	
	LDP2C	
	LDP2D	
	LDP2E	
	LDP2G	
	LDP2H	
	LDP2I	
	LDP2J	
8	23	**Total** (n=31)

Supplementary Table S2. ASV taxonomic classification and relative abundance for each sampled larva. Larvae were sampled from a laboratory culture and from the Mutha River. This Table is presented in an accompanying excel file.

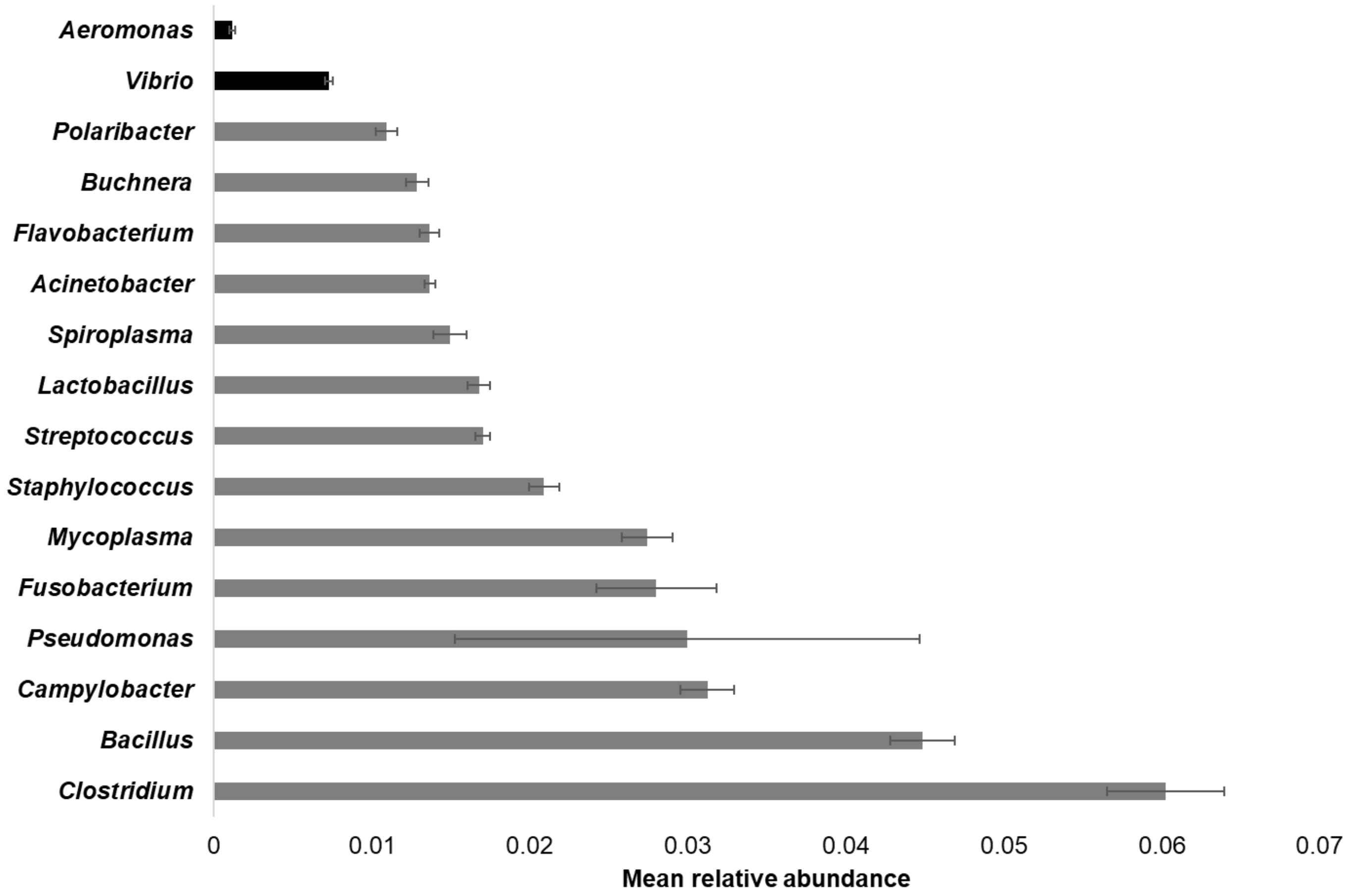

Supplementary Figure S1. The most abundant genera (over 1% of the reads, except for *Vibrio* and *Aeromonas*) **across all samples.** Results are from the metagenomic data of the three larval samples from the Mutha River.
